# Self-reported vs Directly Observed Face Mask Use in Kenya

**DOI:** 10.1001/jamanetworkopen.2021.18830

**Published:** 2021-07-30

**Authors:** Aleksandra Jakubowski, Dennis Egger, Carolyne Nekesa, Layna Lowe, Michael Walker, Edward Miguel

**Affiliations:** 1 Center for Effective Global Action, University of California, Berkeley; 2 Economics Department, University of California, Berkeley; 3 Vyxer Remit Kenya, Busia, Kenya

## Abstract

This cross-sectional study examines the extent to which mask mandates are followed and quantify the bias of self-reported mask usage in Kenya.

## Introduction

Although countries in sub-Saharan Africa have so far avoided large-scale outbreaks of COVID-19, as of June 2021, the number of new cases has been surging in multiple nations. The official case count in Kenya was 179 876 confirmed cases in June 2021, including 1597 cases in Siaya county.^1^ One explanation for the relatively low spread of COVID-19 is the strict lockdown measures that were imposed early but were eventually eased because of their harsh socioeconomic consequences.^2^ Masks emerged as a prominent strategy to reduce SARS-CoV-2 transmission before widespread vaccinations and have been legally mandated in Kenya since April 2020.^3^ Yet compliance with public health guidelines is not guaranteed and motivating people to adopt new preventive health behaviors is inherently difficult. In this cross-sectional study, we examine the extent to which mask mandates are followed and quantify the bias of self-reported mask usage.

## Methods

Ethical approval for this cross-sectional study was obtained from Maseno University Ethics Review Committee and the Committee for Protection of Human Subjects at the University of California, Berkeley. Informed consent was obtained from survey respondents and waived by both institutional review boards for direct observations. This study followed the Strengthening the Reporting of Observational Studies in Epidemiology (STROBE) reporting guideline.

We use data from Ugunja subcounty of Siaya county in Kenya, which is located on a major trade route near the border with Uganda. We observed public behavior including the possession and use of masks, location, situation, gender, and estimated age category in 71 randomly selected villages and 10 weekly markets for 3 hours per day from August 28 to September 11, 2020. We conducted phone interviews from June 18 to September 2, 2020, with male and female respondents who were randomly selected from a 2019 village-level census.^4^ We calculated the proportion of people observed using masks (ie, masks were visible and worn over mouth and nose) and the proportion of respondents self-reporting wearing masks (ie, always or sometimes wears mask in public places). We compared the self-reported and directly observed proportions using regression with survey weights and standard errors clustered at the village and market (the Supplement). Statistical tests were 2-tailed, and significance was set at α = .05. Analyses were performed in Stata 15 (StataCorp).

## Results

We observed the mask behavior of 9533 individuals (5478 [57.5%] men; 5206 [54.6%] individuals in aged 26-45 years). We collected survey data from 1960 individuals who self-reported mask use at a total of 6225 public outings (4251 of 6225 [68.3%] women and 2539 of 6225 [40.7%] aged 26 to 45 years). We documented a large and statistically significant discrepancy between self-reported and observed mask use. We found that 4685 individuals (75.9%) self-reported always wearing a mask in public and 748 individuals (11.7%) self-reported sometimes wearing masks in public. However, 448 individuals (4.7%) were observed wearing a mask correctly in public and another 575 individuals (5.7%) carried a mask (Table). In other words, while only 12% of people admitted to not wearing a mask, 90% were observed not using them (77.7% difference; 95% CI, 80.9%-74.5%).

**Table.** Self-reported vs Direct Observation Mask Use

Characteristic	Observed mask use in public places^a^				Self-reported mask use at public outings^b^				Self-reported vs observed mask use, percentage point difference (95% CI)^c^
	Total No.	No. (%)			Total No.	No. (%)			Always/sometimes vs worn/visible
		Worn correctly	Visible	None		Always	Sometimes	Never	
Full sample	9533	448 (4.7)	545 (5.7)	8540 (89.6)	6225	4685 (75.9)	748 (11.7)	792 (12.4)	77.2 (74.2-80.2)
Gender									
Male	5478	253 (4.6)	252 (4.6)	4973 (90.8)	1974	1467 (74.7)	219 (12.7)	288 (12.6)	78.2 (74.2-82.1)
Female	3822	186 (4.9)	274 (7.2)	3362 (88.0)	4251	3218 (76.4)	529 (11.3)	504 (12.3)	75.7 (71.9-79.5)
Age									
19-25	2415	53 (2.2)	104 (4.3)	2258 (93.5)	123	91 (79.6)	15 (10.7)	17 (9.8)	83.7 (76.5-91.0)
26-45	5206	264 (5.1)	296 (5.7)	4646 (89.2)	2539	1922 (76.5)	309 (11.8)	308 (11.7)	77.6 (73.7-81.5)
46-60	1503	106 (7.1)	114 (7.6)	1283 (85.4)	1253	945 (75.3)	139 (11.1)	169 (13.6)	71.8 (65.8-77.7)
>60	358	23 (6.4)	27 (7.5)	308 (86.0)	844	636 (76.2)	91 (11.3)	117 (12.5)	73.5 (66.7-80.3)
Location									
Market	1397	87 (6.2)	168 (12)	1142 (81.7)	1650	1506 (91.7)	113 (6.5)	31 (1.8)	63.5 (57.0-69.9)
Public transport	995	151 (15.2)	96 (9.6)	748 (75.2)	907	735 (81.9)	60 (6.4)	112 (11.7)	79.9 (72.9-87.0)
Village	6002	128 (2.1)	496 (3.9)	5642 (94)	1255	808 (65.7)	226 (18.0)	364 (16.3)	77.7 (74.5-80.9)
Situation									
Socializing	1593	57 (3.6)	131 (8.2)	1405 (88.2)	NA	NA	NA	NA	NA
Alone	5818	188 (3.2)	284 (4.9)	5346 (91.9)	NA	NA	NA	NA	NA
Commuting	993	151 (15.2)	96 (9.7)	746 (75.1)	NA	NA	NA	NA	NA

^a^ Observed mask use is the proportion of people: (1) wearing masks correctly (over mouth and nose), (2) having masks visible, and (3) having no mask visible. Gender, age (in categories), and situation were estimated by trained enumerators from a distance.

^b^ Self-reported mask use is the proportion of people reporting: (1) always wearing masks, (2) sometimes wearing masks, (3) never wearing masks. Self-reported data are weighted by the probability of being selected for the survey. Phone data were transformed so that each observation represents a respondent’s outing to a public place. Observations with missing data are omitted from analysis.

^c^ Statistical differences in self-reported vs observed mask use were tested by comparing the proportion of people disclosing they always or sometimes use masks vs the proportion of people observed wearing or having masks visible using ordinary least squares regression with standard errors clustered at the village/market level, where each row is a separate regression conditional on the descriptive characteristic.

The discrepancy between self-reports and observations persisted when we examined mask use by gender, age, and location (Figure). The proportion of correct mask use was significantly higher on public transportation (15.2%) than at markets (8.9% reduction; 95% CI, 13.9-4.0) or in villages (13.0% reduction; 95% CI, 16.7-9.3).

**Figure.**
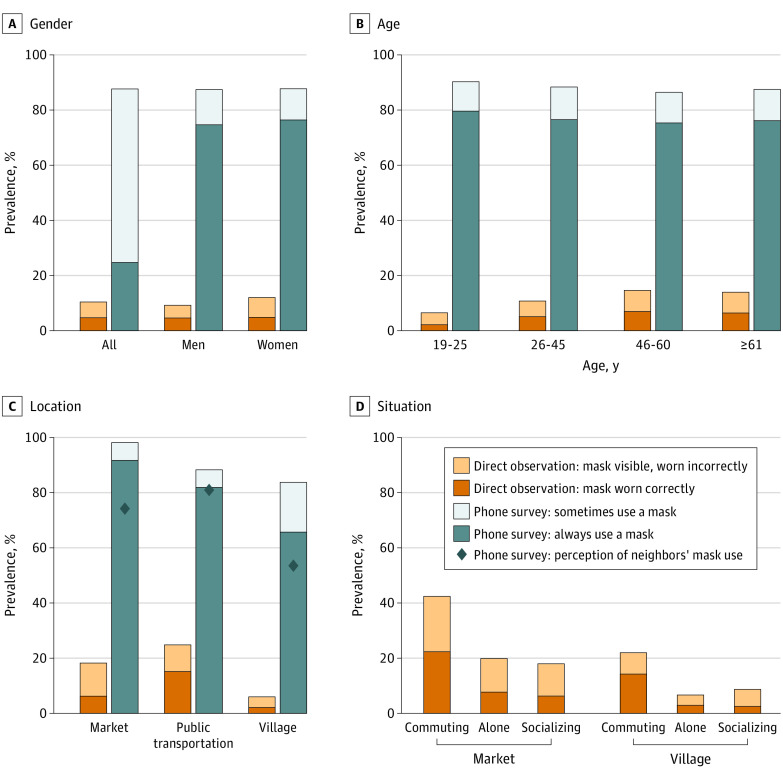
Large Discrepancy in Self-reported vs Direct Observations of Mask Use Persists When Analyzed by Gender, Age, and Location Proportions estimated on sample of 1960 respondents who had been to a public place in the past 7 days and 9553 observations conducted in 71 villages and 10 market centers. Phone surveys were weighted by the probability of being selected for the phone interviews. Approximately 40% of the observed people were women vs almost 70% of the phone respondents were women. Panel B is restricted to participants with age data; no statistically significant differences found in mask use between participants with and without age data. Age and gender in direct observations were estimated by trained enumerators. Panel C also displays the respondents’ perceptions of mask use by their neighbors, which is represented by the diamond markers. Observations with missing data were omitted from analysis.

## Discussion

Masks are among the few tools available to slow the spread of SARS-CoV-2 without imposing major economic hardship before widespread distribution of vaccines. In this cross-sectional study, we found very limited compliance with a national masks mandate in Kenya; only 10% of observed people used masks, which is on par with a finding from Bangladesh.^5^ Conversely, a high proportion of participants (88%) self-reported wearing masks. This discrepancy suggests that although most people are aware that masks are mandated, they have not adopted this new health behavior. Studies relying solely on self-reported mask use may suffer from bias and should be interpreted with caution. This study had limitations, including use of cross-sectional data, estimating age and gender of observed individuals, and the assumption that most observed individuals are from the study area.

Our findings suggest that an increased focus on encouraging mask use is urgently needed in Kenya. Mask policies are already enforced with harsh fines in Kenya, so alleviating barriers to use may offer a more promising strategy. Because people increased mask use in higher-risk situations, reinforcing information about COVID-19 and masks effectiveness may be especially successful. Campaigns that appeal to social desirability of masks, either through modeling behaviors or stressing altruistic motivations,^6^ may be particularly relevant in the context of mask behavior, which inherently involves social signaling.

## Notes

Supplement. eMethods. **eFigure 1. **Timeline of COVID-19 in Kenya eReferences.
