# Supplementary material for: Self-reported vs Directly Observed Face Mask Use in Kenya
Source: JAMA Netw Open. 2021 Jul 30;4(7):e2118830. doi: 10.1001/jamanetworkopen.2021.18830 (PMC8325070; doi:10.1001/jamanetworkopen.2021.18830)
Supplement: Supplement. — eMethods. eFigure 1. Timeline of COVID-19 in Kenya eReferences. [file jamanetwopen-e2118830-s001.pdf]

Supplemental Online Content

Jakubowksi A, Egger D, Nekesa C, Lowe L, Walker M, Miguel E. Self-reported vs directly observed face mask use in Kenya. *JAMA Netw Open*. 2021; 4(7):e2118830. doi:10.1001/jamanetworkopen.2021.18830

eMethods.

eFigure 1. Timeline of COVID-19 in Kenya

eReferences.

This supplemental material has been provided by the authors to give readers additional information about their work.

© 2021 Jakubowski A et al. *JAMA Network Open*.

eMethods.

Study setting

The first case of COVID-19 in Kenya was detected on March 13, 2020 (Figure 1). Within days, President Uhuru Kenyatta restricted travel from other countries, ordered non-essential government and business employees to work from home, and imposed limits on group gatherings. Schools were closed by March 20th and further restrictions (including a ban of international flights, dusk to dawn curfew, and requirement to end dine-in services) were imposed by March 25th. As in many other countries, the restrictive lockdown measures took a harsh socio-economic toll on the population, including falling incomes, increased food insecurity, and increased domestic violence.[1,2] In response, lockdowns were gradually eased in favor of promoting the less invasive mitigation strategies of hand washing, mask use, and physical distancing. Starting in early April, masks have been legally mandated in public spaces in Kenya. By July, restrictions on movement in and out of cities were lifted and international flights resumed in August 2020, though curfews remain in effect. The relaxation of travel restrictions has placed greater importance on COVID-prevention measures in rural areas. After multiple waves of COVID-19,[3] officials in rural settings are becoming increasingly concerned about the spread of the virus there.

eFigure 1. Timeline of COVID-19 in Kenya

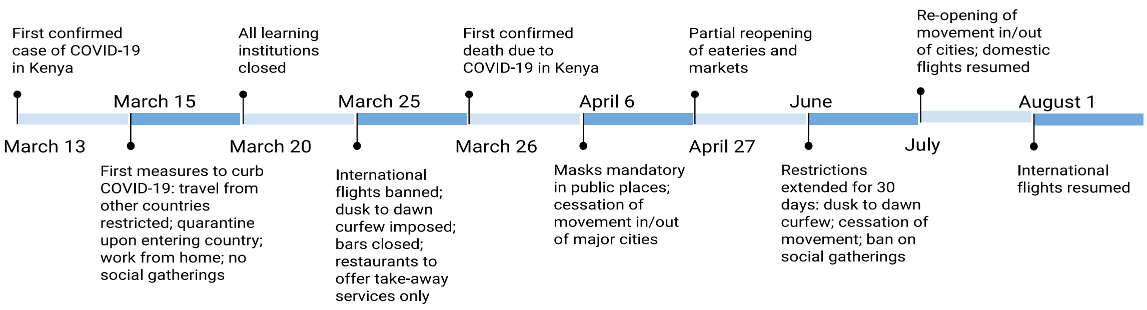

© 2021 Jakubowski A et al. *JAMA Network Open*.

Data Collection Procedures

Data were collected in Ugunja subcounty, located in Siaya County in Western Kenya. This fairly typical rural African setting sits on a major trade route near the border of Kenya and Uganda. We conducted direct observations between August 20 to September 11, 2020, in 71 randomly selected villages and 10 weekly markets. Each village and market were observed for three 1-hour intervals spread over the course of a day. Trained enumerators were instructed to stand at public places and observe behavior related to maintaining physical distancing between people, possession of masks, and proper mask use (i.e., wearing mask over mouth and nose). Common observation places included standing by the side of the road, standing by a shop in the village or between shops in the market, and near public transportation pick-up/drop-off locations. No private information about subjects was collected during direct observations. Public information such as gender, estimated age, activity, and setting type were recorded. The observations were conducted in public spaces without the knowledge of the general public that they were being observed to avoid introducing bias to their behavior.

Household socio-economic surveys were conducted by phone with respondents in Ugunja between June 18 to September 2, 2020. Respondents were randomly selected for participation in the survey from the full set of people living in 166 rural villages in Ugunja, including all 71 villages randomly selected for observations, based on census data collected by members of the research team in late 2019.[4] The surveys gathered information on household characteristics, labor and education, consumption, travel patterns, health, and COVID-19 knowledge and behaviors. We gathered information about masks in the COVID-19 module, including mask ownership, type, and whether respondents wore masks when last visiting public places such their village, markets, religious gatherings, on public transportation, when visiting a shop, or visiting another household. Here, we focus on self-reported mask use in public places that could be compared with direct observations:

© 2021 Jakubowski A et al. *JAMA Network Open*.

villages, markets, and public transportation. Expecting some level of bias in self-reports, we also asked respondents to estimate mask wearing behavior of other people by asking them to estimate the proportion of other households in their village wearing masks to the same public places.

The study procedures were approved by the Institutional Review Boards in Kenya (Maseno University) and the United States (University of California, Berkeley).

Outcome measures

Using direct observations, we constructed three measures of mask use: 1) whether masks were in possession (i.e. visible), 2) whether masks were worn correctly (i.e. covered mouth and nose), and 3) whether no mask was visible to the observers. Using phone surveys, we asked respondents whether they had been to a public place in the past 7 days, and if so, whether they used masks in the specific places they visited. We classify both direct observations and phone survey self-reports by location: within the village, in market centers, on public transport, and other. We then transformed the individual-level phone survey data into person-place observations and constructed three measures of self-reported mask use: 1) always wears masks, 2) sometimes wears masks, and 3) does not wear masks. Respondents who said they never wore masks in public were coded as not using mask in each specific location. Self-reported responses were limited to participants who disclosed they had been to a public place in the week prior to interview. For simplicity, when comparing self-reported and observed data, we group the observed outcomes to a single indicator of whether masks were worn or visible and group the self-reported outcomes to a single indicator of always or sometimes wears masks. We also calculated the estimated proportion of other households wearing masks to the same public places.

© 2021 Jakubowski A et al. *JAMA Network Open*.

Statistical analysis

We calculated the average mask use in the observed and self-reported samples, stratified by age, gender, and location type. We then used linear regression to test for differences between self-reported vs. directly observed mask use by pooling data across samples and including an indicator for data type. Separately, we also tested whether mask use varied by gender, age, and setting. We explored the direct observations further by grouping observations into three broad categories of activities based on risk of virus transmission: 1) commuting activities, primarily (90%) for-hire motorcycles that require sitting in close proximity to the driver, or bus; 2) solitary activities, including working alone in the field, walking/cycling alone, sitting/resting alone; and 3) social activities, including shopping and talking. Similar measures were not possible to construct with phone data. All linear regression models had standard errors clustered at the village/market and were weighted by the sampling probability of being selected for the phone survey to make reported averages representative of the study area population. We checked the robustness of our findings by restricting the phone survey data to the same dates and the specific villages where observations were conducted.

© 2021 Jakubowski A et al. *JAMA Network Open*.

eReferences.

1. Kenya COVID-19 Economic Tracker. https://www.kenyacovidtracker.org. Published 2020. Accessed.
2. Dennis Egger EM, Shana S. Warren, Ashish Shenoy, Elliott Collins, Dean Karlan, Doug Parkerson, A. Mushfiq Mobarak, Günther Fink, Christopher Udry, Michael Walker, Johannes Haushofer, Magdalena Larreboure, Susan Athey, Paula Lopez-Pena, Salim Benhachmi, Macartan Humphreys, Layna Lowe, Niccoló F. Meriggi, Andrew Wabwire, C. Austin Davis, Utz Johann Pape, Tilman Graff, Maarten Voors, Carolyn Nekesa, Corey Vernot. Falling living standards during the COVID-19 crisis: Quantitative evidence from nine developing countries. *Scientific Advances.* 2021.
3. World Health Organization. WHO Coronavirus Disease (COVID-19) Dashboard. https://covid19.who.int/region/afro/country/ke. Published 2020. Accessed.
4. Egger D, Haushofer J, Miguel E, Niehaus P, Walker MW. *General equilibrium effects of cash transfers: experimental evidence from Kenya.* National Bureau of Economic Research;2019.

© 2021 Jakubowski A et al. *JAMA Network Open*.
